# Should pregnant women be excluded from a community-based lifestyle intervention trial? A case study

**DOI:** 10.1186/s12978-017-0422-2

**Published:** 2017-12-14

**Authors:** Elezebeth Mathews

**Affiliations:** Department of Public Health and Community Medicine, Central University of Kerala, Kasaragod, Kerala, India

**Keywords:** Fair inclusion, Lifestyle modification, Type 2 diabetes mellitus, Ethics, Pregnancy research, Clinical translational research

## Abstract

Kerala, the southernmost Indian state, is known as the diabetes capital of the country. A community-based lifestyle modification program was implemented in the rural areas of Kerala, India, to assess effectiveness in reducing the incidence of type 2 diabetes mellitus (T2DM) among individuals at high risk. High-risk individuals for T2DM were identified through home screening and enrolled into the program after an oral glucose tolerance test to rule out T2DM. Pregnant women were excluded from participation in the trial without justification. An analysis is offered to show that exclusion in this case compromised the ethical requirements of fairness and favorable risk-benefit ratio: specifically, pregnant women were deprived of the benefits of screening for high-risk status and subsequent potential involvement in the lifestyle modification intervention, an effective preventive strategy. Exclusion of pregnant women from translational and implementation research with known benefits over risk violates several ethical principles and further limits the exploration and advancement of research for future disease prevention in the population at large. Clearer guidelines on minimal risk and benefit need to be established in order to facilitate research that is beneficial to pregnant women and the developing fetus.

## Background

The occurrence of gestational diabetes mellitus (GDM) is increasing worldwide [1]. As last reported in 2004, the overall prevalence of GDM in India was 16.55% [2], with the highest percentage (15–21%) reported in the state of Kerala [3]. India has the second largest number of individuals with type 2 diabetes mellitus (T2DM), which is expected to double by 2030 [4]. The highest prevalence of T2DM, up to 20% is reported in the state of Kerala [5]. In India, prediabetes and diabetes affect around six million births each year, largely due to GDM (5 million women per year) [6, 7].

Compelling evidence suggests that GDM causes both long- and short-term health effects for the pregnant woman, her fetus, and future child [8–11]. Further, limited evidence suggests that girls born to women with GDM have a higher likelihood of developing GDM themselves [12], causing a vicious trans-generational cycle wherein diabetes begets diabetes.

Although GDM and T2DM are different types of diabetes affecting individuals at different points in the life course, both share common lifestyle risk factors and are responsive to the same prevention and management strategies to a great extent [13–18]. Lifestyle modification is proven to be effective in reducing the incidence of GDM [14] as well as T2DM by 60% among high-risk individuals [15–18].

A cluster-randomized controlled trial of a lifestyle intervention program that compared lifestyle intervention versus health information through a booklet was implemented in 60 voting booths of a *taluk* (sub-district) in Thiruvananthapuram District, Kerala. The trial, funded by an international agency and implemented in collaboration with a local medical institute, involved a preliminary needs assessment in the local community among the target groups. The trial aimed to estimate the effectiveness of a culturally-adapted lifestyle intervention in reducing the incidence of T2DM among high-risk individuals [19]. High-risk individuals (normoglycaemic and prediabetic), based on diabetic risk score, were enrolled into a year-long lifestyle modification program following administration of an oral glucose tolerance test to rule out T2DM. Pregnant women were excluded from the trial. The intervention was a peer-led lifestyle modification program that focused on 1) increasing consumption of fruit and vegetables, 2) increasing physical activity through walking, exercise, and culturally appropriate activities, 3) setting realistic goals and associated targets for weight loss and other lifestyle risks, and 4) reducing alcohol consumption and tobacco use, if any.

## Ethical discussion

Given the disproportionate rise in the burden of chronic diseases in India, especially T2DM, excluding pregnant women certainly seems like a lost opportunity, both for pregnant women who might have benefited directly from the intervention and the general population of women who could have benefited from improved evidence for effective lifestyle interventions against diabetes during pregnancy. Why were pregnant women excluded, and what reasons might be offered to support inclusion?

First, current country-level research ethics guidelines in India encourage exclusion by default. The Indian Council of Medical Research (ICMR) Ethical Guidelines for Biomedical Research on Human Participants [20], considers pregnant women as a special group that thus requires special protection in the research context. Unfortunately, these guidelines offer no criteria or definition for what constitutes an appropriate threshold of minimal risk and benefit for the pregnant women and the fetus, nor do the guidelines specify the types of studies in which pregnant women, as a special population, could be involved. Without clear criteria for inclusion, the default interpretation of the country guidance is to exclude pregnant women.

International guidelines offer greater specification, but these are not consistently applied. The Council for International Organizations of Medical Sciences (CIOMS) states that “for research interventions or procedures that have no potential individual benefits for pregnant and breastfeeding women, the risks must be minimized and no more than minimal, and the purpose of the research must be to obtain knowledge relevant to the particular health needs of pregnant or breastfeeding women or their fetuses or infants” [21]. The CIOMS criteria can be applied to the inclusion of pregnant women in this study, even if adopting a presumption of no potential benefit to pregnant women. Lifestyle modifications are generally non-invasive, though the risk to a pregnant woman and her fetus varies with the intensity of the intervention, its targets, and its strategies. However, this community-based, peer-led lifestyle intervention program would be considered a low-intensity intervention consistent with other guidelines on healthy prenatal behaviors, and therefore does not impose greater than minimal risk to the pregnant woman or fetus.

Second, it is possible that the benefits to pregnant women were not clearly considered. Participation of pregnant women in the trial might have facilitated better health outcomes for the woman, her fetus, and future child. Given the proven benefits of lifestyle modification in delaying or preventing GDM and T2DM among pregnant women, minor modifications to the intervention program to accommodate pregnant women would have provided a reasonable alternative to absolute exclusion. Doing so would also have led to better understanding of the optimum levels of intervention required for diabetes prevention among pregnant women. Specifically, it also could have contributed to a better understanding of the short- and long-term effects of lifestyle modifications during pregnancy, which causes known metabolic changes in women. Furthermore, the inclusion of high-risk pregnant women would not have affected the primary study outcome because GDM in pregnant women is caused not by the state of pregnancy itself, but by the presence of risk factors for diabetes which leads to subsequent high-risk status.

Third, it is possible that widely held but erroneous beliefs about diet and pregnancy led to exclusion. In India, it is broadly believed that pregnant women should consume high-calorie, energy-dense food and restrain from any form of physical activity. The special diet is intended to meet the needs of two—the pregnant women and the growing fetus—and physical activity is thought to cause loss of pregnancy [22]. These myths and taboos increase the risk of pregnant women developing GDM or T2DM thereafter. The prevailing community perspectives on pregnancy pose difficulty to including pregnant women with no “visible health problem” in a trial. There may have been a concern that the community could attribute any complications that might arise during pregnancy to the trial (especially as internationally-funded trials are viewed suspiciously following a previous incident [23]). These beliefs could have been addressed through rigorous consent and community engagement processes; pregnant women could have readily been given this background information and offered the opportunity to make an informed decision [24]. Engaging the wider community and potential participants in rigorous discussions on the under-appreciated risks of diabetes and pregnancy would offer valuable public health education and might address widespread misunderstandings about diet and pregnancy related to cultural practices.

Further, including pregnant women in this study on adapted lifestyle interventions would have offered valuable data to counter prevalent myths and taboos in the community about appropriate diets for pregnant women. Failing to engage the community with the most recent and robust evidence on diabetes risks during pregnancy and effective interventions perpetuates the current knowledge gap in research evidence on the role of lifestyle modification in the prevention of T2DM among pregnant women in India. This could have informed a much-needed public health campaign targeting pregnant women. Finally, empowering the community and pregnant women to weigh the benefit to the woman, fetus, and/or future child against the risk of participation would encourage women to take a more active role in their own health during pregnancy with community support [25].

Fourth, possible practical challenges surrounding the recruitment and retention of pregnant women could have been addressed. There are often challenges surrounding loss to follow-up due to transient migration of pregnant women to their mother’s house for maternal care. There are now innovative approaches to following highly mobile and transient research populations using GPS and mobile technology to facilitate inclusion in research.

There were also reasonable alternatives to excluding *all* pregnant women by using more precise inclusion and exclusion criteria and study monitoring. The team could have adopted a screening tool that is valid for pregnant women, enrolled pregnant women until the first trimester of pregnancy and then monitored pregnant women—assessing their risk status at the third trimester—with the possibility of withdrawing from the study at any time but particularly if there was any concern. Primary exclusion could have been restricted to pregnant women with high blood glucose levels suggestive of gestational diabetes as per the standard criteria.

In light of this more detailed evaluation of the risks and benefits of the study, it seems a clear case of unjustified exclusion for pregnant women. Excluding the entire class of pregnant women from any research, but particularly research that offers the prospect of direct benefits and no foreseeable risks for both mother and fetus, is deeply unjust. Simply by virtue of being pregnant and therefore deemed part of a special population, women in India are being systematically denied access to fair distribution of potentially beneficial research.

## Conclusions

Unjustified exclusion of pregnant women from research, especially from translational and implementation research where there are typically data supporting efficacy, deprives pregnant women from the fruits of research and further limits the exploration and advancement of research on future disease prevention in the greater population.

In India, no clear country-level guidance is offered for determining minimum risk and benefit for the inclusion of pregnant women in research. As a result, most pregnant women are excluded from participating in any kind of research by default. Greater clarity is needed in the national and international ethical guidelines to describe the types of research studies in which pregnant women could potentially participate, such as epidemiological, translational, or implementation research where participation presents clear benefit over risk. Proactively addressing the cultural, ethical, and practical concerns about inclusion of pregnant women in research, and offering examples of appropriate study designs, would begin to correct the injustice of systematic exclusion, and by doing so, offer more meaningful choices to pregnant women for enrolling in potentially beneficial research and promote the development of evidence-based interventions for use during pregnancy.

## Abbreviations

CIOMS: Council for International Organizations of Medical Sciences
GDM: Gestational diabetes mellitus
ICMR: Indian Council of Medical Research
T2DM: Type 2 diabetes mellitus
